# Two case reports of rare Down’s syndrome during in vitro fertilization and embryo transfer (IVF-ET)

**DOI:** 10.1097/MD.0000000000037872

**Published:** 2025-01-31

**Authors:** Parisa Taherzadeh Boroujen, Mitra Nemati, Fatemeh Naderipour

**Affiliations:** a Shahid Beheshti University of Medical Sciences, School of Medicine, Tehran, Iran.

**Keywords:** case report, Down syndrome, in vitro fertilization

## Abstract

**Rationale:**

Despite the potential risks, assisted reproductive technology has provided hope and opportunities for individuals and couples struggling with infertility to conceive and have children. This study presents a case report that describes an occurrence where a pregnancy achieved through in vitro fertilization (IVF) and embryo transfer had to be ended because of the presence of trisomy 21 syndrome.

**Patient concerns:**

Two 26-year-old women who were diagnosed with primary infertility due to polycystic ovary syndrome manifested with overweight and hirsutism.

**Diagnosis:**

These 2 cases were regarded as a diagnosis of Down syndrome, which resulted in the decision to legally terminate the pregnancy when the mother was, in her 12th week and 4 days of gestation. Upon examining the makeup of the cells in the chorionic villi it was discovered that all cells had a chromosomal composition of 47, XX.

**Interventions:**

In these case reports, 2 26-year-old woman with polycystic ovary syndrome underwent assisted reproductive technology and IVF to conceive. The first IVF transfer was unsuccessful, but the second attempt resulted in a successful transfer in both cases. However, a positive screening for Down syndrome led to a legal abortion at 12 + 4 weeks gestation.

**Outcomes:**

Genetic counseling revealed no family history of genetic diseases, and the couple opted for IVF without preimplantation genetic testing. During the trimester of their pregnancies the expectant mothers were initially screened at 12 + 4 weeks after conception. Nuchal translucency examination showed thickening of the fluid at the back of the fetal neck. Moreover, there was an increase in the levels of pregnancy related plasma protein and β human chorionic gonadotropin.

**Lessons:**

These 2 cases underscore the significance of genetic counseling and prenatal screening for couples who are undergoing assisted reproductive technologies, with the purpose of detecting and effectively addressing any possible genetic abnormalities that may arise in their progeny.

## 1. Introduction

The arrival of Louise Brown in 1978 marked an achievement in the first attempt at in vitro fertilization (IVF), which opened the doors to a new era where assisted reproductive technology (ART) has made it possible for many infants to be born. With the advancements in ART, we are seeing a rise in the utilization of techniques such as, IVF and intracytoplasmic sperm injection.^[1–3]^ While ART has become a widespread alternative for treating human infertility, it is unclear if IVF or intracytoplasmic sperm injection increases the risk of congenital disabilities in newborns.^[4,5]^

Aneuploidy, which is the most common genetic abnormality, is considered the leading cause of implantation failure, miscarriage, and congenital disabilities.^[6,7]^ The incidence of aneuploidy in pregnancies resulting from ART is higher in comparison to those resulting from natural conception. This can potentially be attributed to various factors, including maternal age, the utilization of ovarian stimulation, and the environmental conditions provided for embryo culture. Nevertheless, the progression of advanced techniques within ART, such as preimplantation genetic testing, has contributed to the reduction of the risk associated with aneuploidy in ART pregnancies.^[8]^

Despite the potential risks, ART has provided hope and opportunities for individuals and couples struggling with infertility to conceive and have children. This study presents a case report that describes an occurrence where a pregnancy achieved through IVF and embryo transfer (IVF-ET) had to be ended because of the presence of trisomy 21 syndrome. The primary aim of this investigation was to document this exceptional case and emphasize the significance of conducting comprehensive and prolonged monitoring to detect any occurrences of aneuploidy.

## 2. Case 1

Prior to the intervention, couples were required to provide written informed consent for publication of the case. A 26-year-old woman who was diagnosed with primary infertility due to polycystic ovary syndrome manifested with overweight and hirsutism. The spouse of the female, who was 28-years-old, exhibited typical semen quality, and both he and the female possessed regular karyotypes (46, XX and 46, XY) without any noticeable genetic anomalies. To aid her in the process of becoming pregnant, the female underwent ART and IVF, during which 2 embryos were introduced. The first transfer was negative, while the second try resulted in a successful transfer.

**Figure 1.**
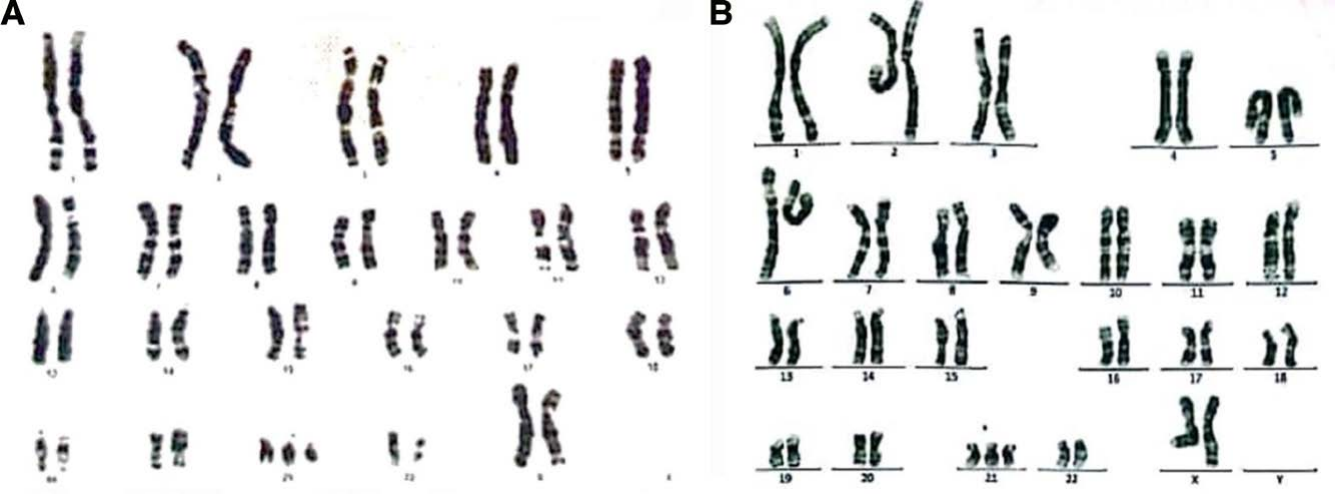
Chromosome analysis compatible with apparently trisomy 21 extracted from amniotic fluid sample. (A) case 1; (B) case 2.

Genetic counseling was conducted, which revealed no family history of genetic diseases, and the couple was advised to undergo IVF-ET without preimplantation genetic testing-A. The IVF procedure followed the standard long protocol, which involved ovarian stimulation, egg retrieval, fertilization in the laboratory, and embryo transfer. The mother underwent first-trimester screening at 12 + 4 weeks gestation. Nuchal translucency (NT) examination which is a type of ultrasound scan that measures the thickness of the fluid at the back of the fetus’s neck revealed NT of 2.3 mm (82 percentile) and the pregnancy-associated plasma protein-A (PAPP-A) and beta-human chorionic gonadotropin (β-hCG) were 1.05 mIU/mL and 48.20 IU/L, respectively. All these numbers were interpreted as a positive screen for Down syndrome resulted in legal abortion when the mother was 12 + 4 weeks pregnant. Chorionic villi were extracted from the products of conception and employed as initial substance for the establishment of tissue cultures. The examination of the karyotype of these cell lines exhibited a chromosomal complement of 47, XX in all analyzed cells (Fig. 1A).

## 3. Case 2

Prior to the intervention, couples were required to provide written informed consent for publication of the case. A pair of individuals, a 26-year-old woman and a 36-year-old man, who lack any familial connection or history of genetic disorders, decided to partake in ART utilizing IVF. The female displayed characteristics of being overweight and experiencing hirsutism, while the male exhibited a normal sperm analysis. Despite both parents having normal karyotypes (46, XX for the mother and 46, XY for the father), a sample of amniotic fluid obtained when the mother was 12 + 6 weeks pregnant revealed the presence of trisomy 21, as evidenced by the trisomy of the 21st chromosome. This discovery prompted the termination of the pregnancy at this stage due to a positive result from the first-trimester screening, which involved measuring the nuchal translucency (NT = 2.7 mm) and assessing the PAPP-A and β-hCG, which were found to be within abnormal ranges at 1090 mIU/mL and 65.2 IU/L, respectively. The examination of the karyotype of chorionic villi exhibited a chromosomal complement of 47, XX in all analyzed cells (Fig. 1B). Since this study is a case report, there was no need for institutional review board approval, however, patients’ information was reported after obtaining informed consent.

## 4. Discussion

In this scenario, a youthful pair with typical chromosomal compositions experienced the process of fertilization outside the body (IVF) and the transfer of the embryo (embryo transfer or ET) after the identification of an extra copy of chromosome 21 (trisomy 21) during the screening conducted in the initial stage of pregnancy. This instance emphasizes the significance of obtaining guidance from experts in genetics and undergoing prenatal examinations for couples utilizing assisted reproductive technologies, to ascertain and address any potential genetic irregularities that may arise in their offspring.

Trisomy 21 is a genetic anomaly that manifests when there is an additional duplicate of chromosome 21 in either all or a subset of the body’s cells. Down syndrome serves as a prevalent illustration of trisomy 21, which is typified by different physical and cognitive impairments. Selected indicators of trisomy 21 encompass cognitive incapacitation, congenital heart anomalies, and muscular hypotonia.^[9–12]^ The risk factors for trisomy 21 include advanced maternal age, environmental factors, and errors during meiosis or mitosis.^[13–16]^

Fetal NT thickness is a quantification of the accumulation of fluid at the posterior aspect of the fetal neck. This measurement is conducted during the initial trimester of gestation and serves as a means of identifying chromosomal abnormalities, notably Down syndrome. Maternal serum biochemical markers, specifically PAPP-A and free β-hCG, are proteinaceous entities that are produced by the placenta and can be identified in the maternal bloodstream. These markers are additionally utilized as a means of screening for chromosomal abnormalities during the initial trimester of gestation. Noninvasive prenatal testing represents a screening methodology that capitalizes on a maternal blood specimen to determine the existence of fetal DNA within the maternal circulation. This examination exhibits a high level of precision in detecting chromosomal abnormalities, including Down syndrome. Noninvasive prenatal testing employs high-throughput methodologies to identify free placental DNA (cfDNA), which denotes the minute fragments of fetal DNA that are present in the maternal blood. These fragments are liberated into the maternal bloodstream by the placenta and can be discerned through specialized laboratory techniques. These prenatal screening modalities are commonly employed to provide a risk assessment for prevalent autosomal aneuploidies, which denote chromosomal abnormalities characterized by an excess or deficiency of chromosomes. Down syndrome serves as an exemplar of an autosomal aneuploidy that can be identified through these screening techniques.

In this report, NT played an important role in diagnosis of the abnormality. Previous studies also demonstrated the importance of the NT alone as a reasonable option that deserves further investigation.^[17]^ In line with the study by Taheripanah et al,^[18]^ PAPP-A levels were lower but free β-hCG levels were higher in this case of IVF. In conclusion, when a healthy couple is referred for IVF, first-trimester screening should be given special attention to detect any abnormalities.

Regarding the limitations, we should mention the case report nature of this study. Case report studies have some common limitations. One limitation is that case reports are often seen as providing exploratory or descriptive evidence, rather than definitive conclusions. Another limitation is the lack of consensus on what constitutes a case study, leading to diversity in how they are conducted and reported. Additionally, case reports typically focus on providing detailed descriptions of individual cases, which can make it challenging to identify key messages related to intervention evaluation. Furthermore, case reports lack statistical sampling, controls, and have limited sample sizes, which can limit the generalizability of their findings. Finally, case reports may be subject to publication bias, as journals may be more inclined to publish case reports that provide new information on adverse events or interventions.

## Author contributions

**Data curation:** Fatemeh Naderipour.

**Methodology:** Mitra Nemati.

**Writing – original draft:** Fatemeh Naderipour, Mitra Nemati.

**Writing – review & editing:** Parisa Taherzadeh Boroujen.
